# Synthesis of the Novel Covalent Cysteine Proteases Inhibitor with Iodoacetic Functional Group

**DOI:** 10.3390/molecules25040813

**Published:** 2020-02-13

**Authors:** Kinga Hartman, Przemyslaw Mielczarek, Jerzy Silberring

**Affiliations:** 1 Department of Biochemistry and Neurobiology, Faculty of Materials Science and Ceramics, AGH University of Science and Technology, Mickiewicza 30, 30-059 Krakow, Poland; kinga.piechura24@gmail.com (K.H.); jerzy.silberring@agh.edu.pl (J.S.); 2 Polish Academy of Sciences, Maj Institute of Pharmacology, Laboratory of Proteomics and Mass Spectrometry, Smetna 12, 31-343 Krakow, Poland

**Keywords:** iodoacetic acid, solid phase synthesis, DCG-04, inhibitor, cysteine protease

## Abstract

This work presents the synthesis of the novel covalent inhibitor of cysteine proteases where epoxide has been replaced by the iodoacetyl functional group. The molecule, similar in action to E-64 and DCG-04, the commonly applied inhibitors, is additionally biotinylated and contains tyrosyl iodination sites. The Fmoc solid phase synthesis has been applied. Conjugation of iodoacetic acid with the peptide was optimized by testing different conjugation agents. The purity of the final product was verified by mass spectrometry and its bioactivity was tested by incubation with a model cysteine protease—staphopain C. Finally, it was shown that the synthesized inhibitor binds to the protein at the ratio of 1:1. More detailed analysis by means of tandem mass spectrometry proved that the inhibitor binds to the cysteine present in the active site of the enzyme.

## 1. Introduction

**Figure 1:**
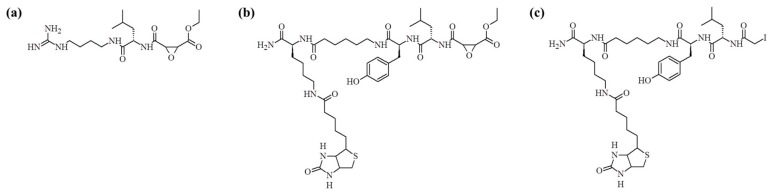
Chemical structures of cysteine proteases inhibitors. (**a**) E-64, (**b**) DCG-04, and (**c**) novel inhibitor.

Proteases are important enzymes that regulate a variety of biological processes in living organisms [1,2,3]. Their activity may be controlled by endogenous inhibitors [4,5]. Such pathways may comprise an attractive target for drug design, and endogenous protease inhibitors may serve as lead compounds to design synthetic analogs [6]. One of the best known inhibitors of cysteine proteases is E-64 [7,8]. This molecule (see Figure 1a) inhibits a broad spectrum of cysteine proteases and its structure is based on the epoxide structure, which allows for its covalent binding to the active site cysteine. As a result, E-64 is an irreversible inhibitor of this class of enzymes. In the work published by Greenbaum et al., the structure of this inhibitor was modified by an introduction of the affinity tag (biotin) leading to the synthesis of DCG-04 (see Figure 1b) [9]. This modification resulted in the design of chemical probes used to monitor cysteine proteases activity. This molecule, however, still contains epoxide electrophile, what makes synthesis quite demanding, especially with an application of the solid phase synthesis. Another well-known cysteine proteases inhibitor is iodoacetic acid and its amide [10]. This molecule also acts as an electrophile and can covalently modify sulfhydryl groups present in the cysteine side chain. This approach is often employed in proteomics after protein reduction, to avoid reversible cysteine oxidation. The reaction also finds applications in protein crosslinking [11], peptide synthesis, and conjugation with other molecules via cysteine side chains [12]. Another interesting utilization of iodoacetic acid is stable isotope labeling of peptides and proteins with ^16^O/^18^O iodoacetic acid, which can also be used in quantitative proteomics [13].

In this paper, we present the synthesis of a new inhibitor of cysteine proteases based on the structure of DCG-04 where the epoxide group has been replaced with the iodoacetyl group. Application of bromo-, chloro-, and iodoacetic acids in the peptide solid phase synthesis requires changes in the standard synthesis protocol [14]. The results demonstrate a comparison of standard synthesis involving DIC (*N*,*N*′-diisopropylcarbodiimide) with HOBt (*N*-hydroxybenzotriazole), with the newer activation reagent like COMU (1-Cyano-2-ethoxy-2-oxoethylidenaminooxy)dimethylamino- morpholino-carbenium hexafluorophosphate) [15,16,17]. Synthesis yield and product purity were checked by mass spectrometry [18]. Inhibitory potency was tested using staphopain C as a reference enzyme [19,20]. The synthesis is much easier and cheaper and does not require any atypical conditions. 

## 2. Results and Discussion

**Figure 2:**
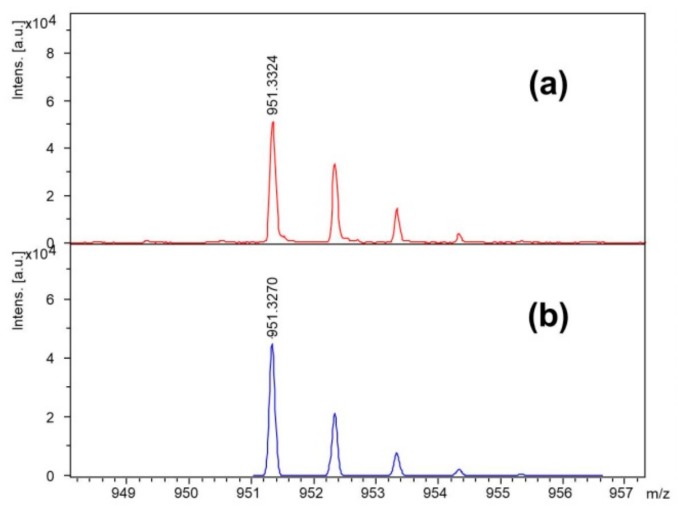
(**a**) High resolution matrix-assisted laser desorption/ionization time-of-flight (MALDI-TOF) mass spectrum of the synthesized IAA-Leu-Tyr-Ahx-Lys(biotin)-NH_2_; sodium adduct was observed as an ion at *m*/*z* 951.3324. (**b**) Theoretical high resolution mass spectrum representing ion C_39_H_61_IN_8_O_8_SNa^+^.

The reaction of iodoacetic acid with peptide immobilized on the resin was carried out using various coupling reagents, as shown in Table 1 in the Materials and Methods. In order to determine the optimal process conditions, the obtained compounds were analyzed using the ESI-MS technique. Chemical composition of the synthetic product was determined based on the high resolution mass spectra presented in Figure 2.

**Figure 3:**
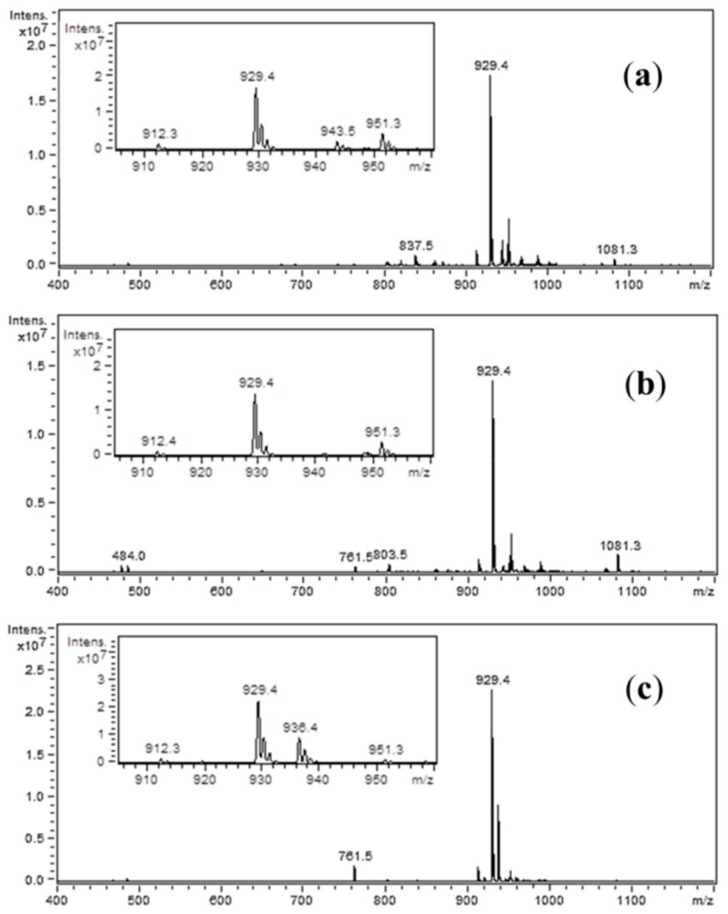
Mass spectra of the synthesized inhibitor (IAA-Leu-Tyr-Ahx-Lys(biotin)-NH_2_), after iodoacetic acid conjugation using several coupling methods: (**a**) COMU/DIPEA; (**b**) DIC; (**c**) DIC/HOBt.

**Figure 4:**
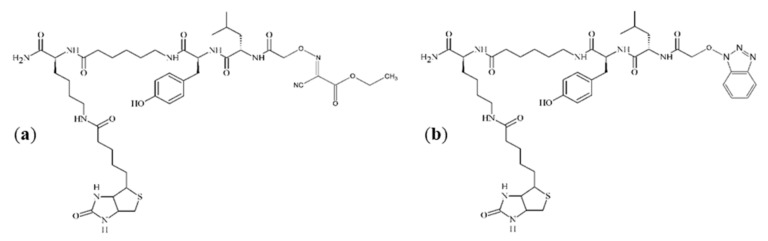
The proposed chemical structures of impurities, visible on the spectrum as ions at *m*/*z* equal to: (**a**) 943.5; (**b**) 936.4.

On the mass spectra (Figure 3), the ion at *m*/*z* 929.4 represents the analyzed compound. In the inner window, the spectra enlargement around this *m*/*z* value is presented. In each case, the peak at *m*/*z* 912.3 is visible. This ion is most likely due to the loss of ammonia molecule. In addition, an ion of *m*/*z* 951.3 can be recognized as a sodium adduct. In Figure 3a,c, additional impurities can be observed. These impurities were formed during peptide synthesis. In the case of spectrum (a), it is an ion at *m*/*z* 943.5, which could be generated by nucleophilic replacement of the iodine by reacting with the ethyl-2-hydroxyimino-2-cyanoacetate moiety that comes from the (1-cyano-2-ethoxy-2-oxoethylidenaminooxy)dimethylamino-morpholino-carbenium hexafluorophosphate (COMU) reagent. An ion at *m*/*z* 936.4, which is presented in spectrum (c), may be formed by nucleophilic replacement of the iodine by reacting with the benzotriazole moiety of HOBt. The proposed structures of these impurities are shown in Figure 4. On the spectra, an ion at *m*/*z* 1081.5 can also be seen. However, due to the presence of this peak on all three spectra, it is suggested that this impurity was not generated by the side-reactions of coupling reagents. When analyzing the spectrum shown in Figure 3b, no additional impurities were observed. This allowed to conclude that, among the coupling reagents used, the DIC used alone proved to be the most advantageous, thus allowing the product to be obtained with the highest yield and purity.

In this study, a novel, synthetic, and biotinylated inhibitor was tested for its ability to bind to cysteine protease in the active site of the enzyme, which would enable its application in this type of investigation. The labeled synthetic cysteine protease inhibitor can be used as a tool to identify cysteine proteases in biological samples, to perform pharmacological experiments, and to be used in activity-based proteomics, to name only a few applications. 

**Figure 5:**
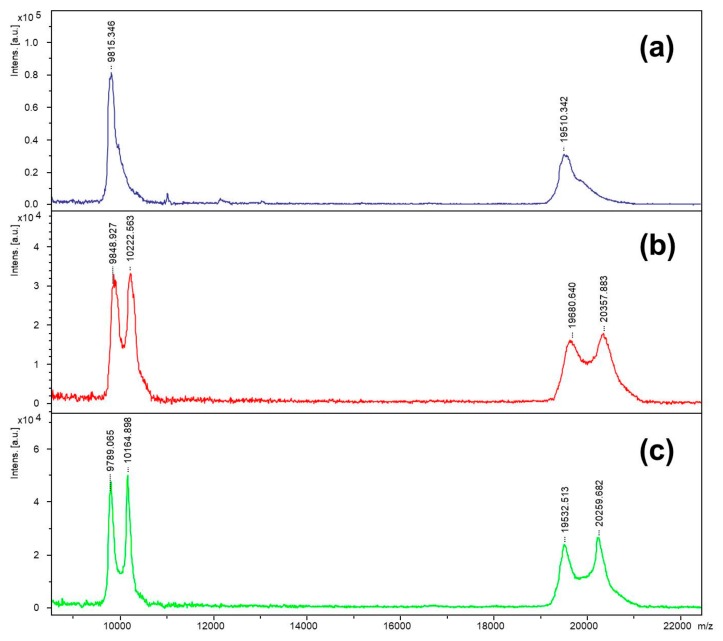
MADI-TOF spectra of (**a**) staphopain C; (**b**) biotinylated inhibitor after incubation with staphopain C at pH 5.0; (**c**) biotinylated inhibitor after incubation with staphopain C at pH 7.8.

The inhibitor was incubated with staphopain C, and the incubation mixture was analyzed by the MALDI-TOF technique in a linear mode. Spectra are shown in Figure 5. Furthermore, the clear signal arising from the enzyme molecule in the mass spectra for both incubation conditions at pH 5.0 and 7.8 showed an additional peak observed at *m*/*z* higher that the peak corresponding to staphopain C. Based on the low resolution MALDI-TOF mass spectra obtained in the linear mode, the difference between peaks for incubation at pH 5.0 was equal to *m*/*z* 373.6, which corresponded to a change in the mass of 747.3 Da (calculation based on the doubly charged ions, to maintain better resolution, which decreases with an increasing *m*/*z* value). For the incubation performed at pH 7.8, the distance between peaks equaled 375.8 *m*/*z*, which corresponds to a change in the mass of 751.7 Da. The distance between the peak and the ion representing the enzyme corresponds to the mass of one molecule of the biotinylated inhibitor covalently attached to the enzyme, after the release of a molecule of hydroiodic acid (the mechanism explained later in the text). This suggests that the obtained inhibitor binds to staphopain C at a ratio equal to 1:1.

**Figure 6:**
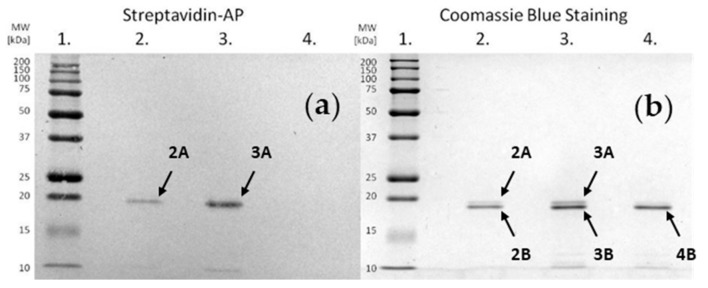
(**a**) Image of the membrane after the electroblotting. (**b**) Image of the gel obtained by sodium dodecyl sulphate polyacrylamide gel electrophoresis (SDS-PAGE) separation. Wells numbered 1–4 correspond to: 1.—MW standards; 2.—staphopain C incubated with biotinylated inhibitor at pH 5.0; 3.—staphopain C incubated with biotinylated inhibitor at pH 7.8; 4.—staphopain C. Bands labeled “A” correspond to staphopain C with a covalently attached inhibitor and bands labeled “B” correspond to native staphopain C.

Electrophoretic separation and electroblotting were carried out as described in the Materials and Methods. The obtained results are presented in Figure 6, showing single bands corresponding to staphopain C. Incubation of the enzyme with an inhibitor at both pHs resulted in the bands located above that of staphopain C, which indicates the formation of the complex. The intensity of the signal representing the product was higher in well No. 3, suggesting a more effective binding at pH 7.8. As a result, the top band in well No. 3 was further analyzed.

**Figure 7:**
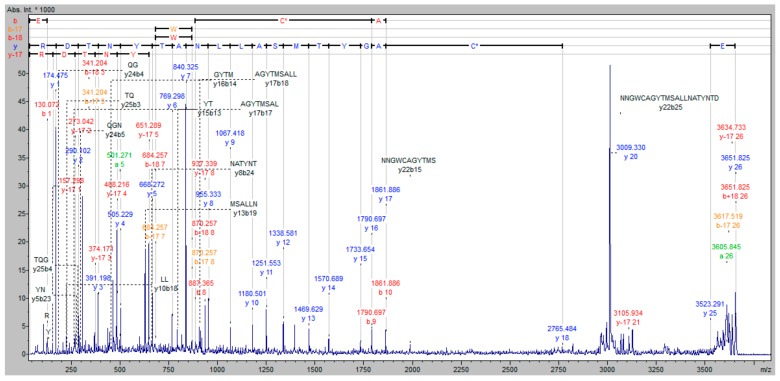
MALDI-TOF/TOF mass spectrum of the peptide (ETQGNNGWCAGYTMSALLNATYNTDR) obtained after tryptic digestion of staphopain C and incubated with a biotinylated inhibitor at pH 7.8. The cysteine modified at its side chain by the reaction with the inhibitor is designated C*.

**Figure 8:**
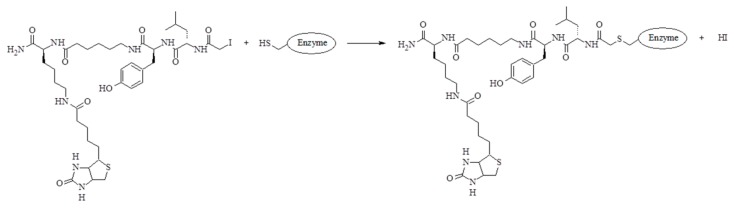
Diagram presenting the mechanism of binding of a biotinylated inhibitor to the active site of cysteine protease.

MALDI-TOF/TOF analysis was first applied to the examination of staphopain C alone. Tryptic peptide with the following amino acid sequence was identified: ETQGNNGWCAGYTMSALLNATYNTDR, corresponding to the partial sequence of the active site of an enzyme, containing Cys. Analyzing the protein band visualized after electroblotting, which is the reaction product between staphopain C and the biotinylated inhibitor at pH 7.8, the spectrum shown in Figure 7 indicates the presence of the peptide with the modified cysteine. The apparent mass difference of cysteine residue corresponds to the weight of one molecule of the biotinylated inhibitor after covalent attachment to the amino acid. On the mass spectrum (Figure 7), the cysteine modified at its side chain by the addition of inhibitor was designated C*. This unambiguously confirms that the process of binding of the obtained inhibitor with the model cysteine protease (staphopain C) takes place in the active site of the enzyme with the release of hydroiodic acid, which is schematically shown in Figure 8.

## 3. Materials and Methods

### 3.1. Chemicals

The reagents from the following companies were used during the experiments: Advanced ChemTech (Louisville, KY, USA): HOBt, Fmoc protected amino acids (Fmoc-AA-OH), with exception of Fmoc-ε-Ahx-OH, Fmoc-Arg(Pbf)-OH, Fmoc-Lys(biotin)-OH, Fmoc-Lys(Mtt)-OH, which were from Novabiochem (Merck) (Warszawa, Poland); Aldrich (Poznan, Poland): hexafluoroisopropanol (HFIP); Bio-Rad (Warszawa, Poland): acrylamide, buffers: Tris-HCl pH 6.8, Tris-HCl pH 8.8, 20% sodium dodecyl sulfate (SDS), buffers: TGS, TG, TBS, TBST, Bio-Safe Coomassie Stain (solution of Coomassie Brilliant Blue G); Fluka (Poznan, Poland): acetaldehyde, p-chloranil, 1-methyl-2-pirolidone (NMP), diizopropylcarbodiimide (DIC); J. T. Baker (WITKO) (Lodz, Poland): methanol, acetonitrile (ACN); Novabiochem (Merck): COMU; Sigma (Poznan, Poland): acetic acid, β-mercaptoethanol, tetramethylethylenediamine (TEMED), ammonium bicarbonate, etylenediaminetetraacetic acid (EDTA); Promega (Warszawa, Poland): trypsin; Sigma-Aldrich (Poznan, Poland): dichloromethane (DCM), dimethylformamide (DMF), *N*,*N*-diisopropylethylamine (DIPEA), trifluoroacetic acid (TFA), phenol, iodoacetic acid, formic acid, acetone, ammonium persulfate (APS); VectorLab (BIOKOM) (Janki, Poland): casein, alkaline phosphate substrate (Vector Black). Water (18 Mohms) used in the studies was purified using the Simplicity system (Millipore) (Warszawa, Poland).

### 3.2. Instrumentation

Purity of the synthesized compounds was tested by means of mass spectrometry, an amaZon ETD ion trap mass spectrometer (Bruker Daltonics, Bremen, Germany) with a standard electrospray (ESI) ion source. The set-up was as follows: capillary voltage of −4 kV, air was used as a nebulizing gas at 3 bar [21], flow rate of drying gas was set to 12 L/min, temperature of the heated capillary was adjusted to 300 °C, and helium was used as a collision gas [22].

For protein identification and determination of the binding site where the inhibitor binds to the protein, chromatographic separation was performed by the nano-flow chromatographic system (EASY-nLC II, Bruker Daltonics) on a capillary reversed phase column C18 equipped with an on-line fraction collector (PROTEINEER fc II, Bruker Daltonics), and the fractions were directly deposited on the MALDI target plates. Samples were subjected to the analysis using a MALDI-TOF/TOF mass spectrometer (ultrafleXtreme, Bruker Daltonics), which allowed us to obtain the maximum sensitivity of determinations as well as high repeatability [23].

### 3.3. Solid Phase Synthesis of IAA-Leu-Tyr-Ahx-Lys(biotin)-NH_2_

**Figure 9:**
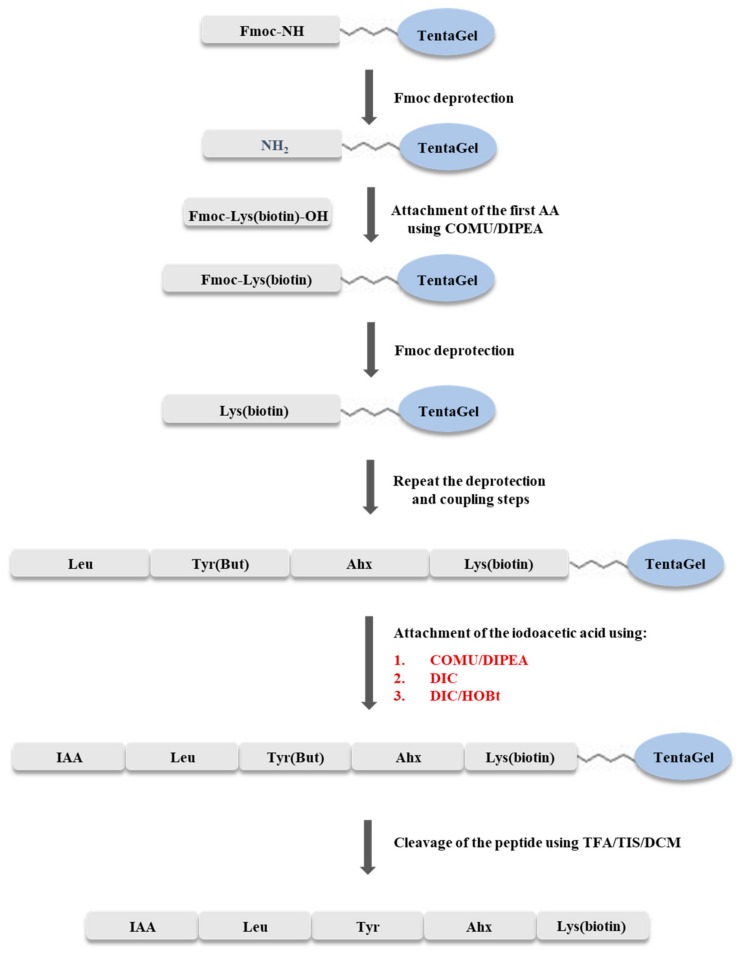
A scheme of the synthesis of IAA-Leu-Tyr-Ahx-Lys(biotin)-NH_2_.

The synthesis of IAA-Leu-Tyr-Ahx-Lys(biotin)-NH_2_ was carried out using the Fmoc strategy on a solid support, according to the scheme shown in Figure 9. The synthesis was carried out manually, in syringes with glass filter, at room temperature. TentaGel S RAM (0.5 g, capacity of the resin 0.23 mmol/g) resin was used for synthesis. The resin was washed with dimethylformamide (DMF) solution for one hour to swell the beads. The Fmoc group was cleaved by washing the resin twice with 20% solution of piperidine in DMF (6 mL), with a wash time of 10 min. Elongation of the peptide chain was accomplished by attaching amino acids blocked at the N-terminus by the Fmoc group. Each coupling step was carried out for two hours, using a triple excess of the amino acid (0.345 mmol) dissolved in DMF (205 mg of Fmoc-Lys(biotin)-OH, 122 mg of Fmoc-ε-Ahx-OH, 159 mg of Fmoc-Tyr(*t*Bu)-OH, 122 mg of Fmoc-Leu-OH), a triple excess of COMU dissolved in DMF (0.345 mmol, 148 mg), and a six-fold excess of DIPEA (0.690 mmol, 121 µL). Exceptionally, when coupling biotinylated lysine, a triplicate excess of the amino acid was dissolved in DCM/NMP (1:2) because of its low solubility in DMF. Completeness of coupling and deprotection was confirmed by a chloranil test. After incorporation of the last amino acid, optimization of the coupling process of the resulting peptide with iodoacetic acid was performed.

Optimization of the synthesis was carried out in three syringes. In the first step, the resins with attached peptides were washed twice with DMF. The Fmoc group was then cleaved twice with 20% piperidine solution in DMF (2 mL). Deprotection time was 10 min. Subsequently, the coupling process with iodoacetic acid was then carried out using the coupling reagents shown in Table 1.

In syringe no. 1, the coupling process was carried out using triple excess of iodoacetic acid (0.069 mmol, 13 mg), triple excess of COMU dissolved in DMF (0.069 mmol, 30 mg), and a six-fold excess of *N*,*N*-diisopropylethylamine (DIPEA) (0.138 mmol, 24 µL). In the second syringe (no. 2), a triple excess of iodoacetic acid (13 mg) and a triple excess of HOBt (0.069 mmol, 11 mg), dissolved in DMF, and a triple excess of DIC (0.069 mmol, 11 µL) were used. In the third sample (syringe no. 3), the coupling was carried out by the addition of a triple excess of iodoacetic acid dissolved in DMF (0.069 mmol, 13 mg), and a triple excess of DIC (0.069 mmol, 11 µL). In each case, the reaction was carried out for two hours. Between the Fmoc cleavage process and iodoacetic acid addition, the peptidyl resin was washed three times with DMF, twice with DCM, and finally twice with DMF.

After the coupling reaction, the resin was washed twice with methanol and DCM. The cleavage of the peptide from the resin was performed simultaneously with the side chain unblocking by the addition of 2 mL of a TFA/TIS/DCM mixture (95/2.5/2.5, *v*/*v*/*v*). The resin was washed twice with this mixture for 30 min (first wash) and 5 min (second wash), respectively. Subsequently, after concentrating the solution with a stream of nitrogen (the total volume was reduced four times at ambient pressure), the products were precipitated with cooled ether, and centrifuged. The precipitate was dissolved in 1 mL 0.1% TFA and then lyophilized. To establish the optimal method of iodoacetic acid attachment, the obtained products were subjected to ESI-MS analysis [21]. The structure of the synthesized compound was confirmed based on high resolution mass spectrometry (HRMS) (see Figure 2) and ^1^H NMR (refer to Supplementary Materials).

### 3.4. Identification of the Binding Site of Biotinylated Inhibitor with Staphopain C

Initially, the incubation process was carried out to bind the biotinylated inhibitor to staphopain C. The reaction was run in parallel at two pH values of 5.0 and 7.8 using acetate buffer (pH = 5.0) or Tris-HCl buffer (pH = 7.8), respectively. Subsequently, staphopain C (3.3 μg) and biotinylated inhibitor (0.1 nmol) were added to each tube. Incubation was carried out at 37 °C for 30 min. In addition, a reference sample was prepared by dissolving the enzyme in water. After the reaction has been completed, termination was performed by means of protein precipitation. An eight-fold excess of cooled (−20 °C) acetone was added to each tube and placed at −20 °C for 15 min. Successively, the samples were centrifuged for 10 min (10,000 g, 4 °C). The supernatant was removed, while the obtained pellet was dried in a vacuum centrifuge. Immediately after drying, portions of the pellets were analyzed by MALDI-TOF using 2,5-dihydroxybenzoic acid (DHB) as a matrix. The rest of the obtained pellets was subjected to gel electrophoresis.

After drying, the precipitate was dissolved in the electrophoresis sample buffer (Laemmli buffer). The resulting solution was incubated at 90 °C for 5 min and then centrifuged at room temperature (10,000 g) for 5 min. The same amount of each sample was loaded into all wells (10 μL of each sample). The samples were separated by the polyacrylamide gel electrophoresis under denaturing conditions (SDS-PAGE). The separation was carried out in 4% stacking gel and 12% resolving gel.

Electrophoresis was performed in the TGS buffer. During the first 15 min of separation, constant voltage of 50 V was applied, and then the voltage was increased to 150 V. After completion of the electrophoretic process, one of the gels was stained and then the separated compounds were prepared for the mass spectrometric analysis, while for the second gel, electroblotting was performed directly after the separation.

The bands containing proteins were prepared for the mass spectrometry analysis [24]. The excised gel fragments were incubated for 10 min at 40 °C in 100 mM ammonium bicarbonate. Subsequently, acetonitrile was added to each tube to reach 50% and incubated for 10 min at 40 °C. The solution was removed and then the incubation step was repeated three times until the dye was completely washed out from the gel. After removing the supernatant, the gel bands were dehydrated by adding anhydrous acetonitrile (ACN). The ACN solution was then removed and the gels were dried in a vacuum centrifuge for 10 min. In the next step, trypsin (0.912 μg of the Promega Gold trypsin in 100 mM ammonium bicarbonate) was added. The reaction was carried out for 45 min at 4 °C and then the gel fragments were covered with 50 mM ammonium bicarbonate and the digestion was carried out at 37 °C overnight. The supernatant, containing peptides resulting from the digestion, was collected and retained for further analysis. The gel was immersed in 50 mM ammonium bicarbonate and incubated at 40 °C for 10 min. Subsequently, ACN was added and incubated at 40 °C for a further 10 min. The resulting supernatant was collected and combined with the previous ones. For further extraction of peptides, the gel was incubated with 5% formic acid in 50% ACN at 40 °C for 10 min. The extraction was carried out twice. The gel fragments were then dehydrated in ACN. The solutions were collected and pooled together with the supernatants obtained previously. Combined samples were dried in a vacuum centrifuge and analyzed by the MALDI-TOF/TOF mass spectrometer. α-cyano-4-hydroxycinnamic acid (CHCA) was used as a matrix.

Electrophoretically separated proteins were transferred from the gel to a polyvinylidene difluoride (PVDF) membrane using wet electroblotting. The PVDF membrane was activated with methanol and rinsed with Tris-Glycine buffer supplemented with 20% methanol. The electroblotting was carried out overnight at a DC current (100 mA) at 4 °C. After electroblotting, the membrane was blocked with casein solution (VectorLab) for 1 hour and then rinsed with the Tris-Buffered Saline (TBS). The membrane was then incubated with a solution containing streptavidin conjugated with alkaline phosphatase. In the next step, the membrane was rinsed twice in Tris-Buffered Saline with 0.05% (*v*/*v*) Tween-20 (TBST) and once in TBS buffer. To visualize the protein bands, 100 mM Tris-HCl buffer (pH 9.5) and an alkaline phosphatase substrate were applied to the membrane [24]. Finally, the membrane was rinsed three times with water, dried, and then documented using the Gel Doc XR+ system from BioRad.

## 4. Conclusions

In the present study, a novel biotinylated inhibitor of cysteine proteases was synthesized using the Fmoc strategy where iodoacetic acid replaced the epoxide moiety. Optimization of iodoacetic acid coupling was performed using three reagents: COMU/DIPEA, DIC, and DIC/HOBt. The efficiency of the reaction was highest using DIC/HOBt and DIC, for which the crude inhibitor was obtained with a yield of 35%. Based on the ESI-MS analysis, it was shown that, among the coupling reagents used, DIC proved to be the best and allowed us to obtain the product with the highest yield and purity. The inhibitor was much simpler to synthesize than those based on epoxides and all reaction steps and intermediates were also stable at ambient temperature. 

The results obtained by the MALDI-TOF/TOF technique showed that the inhibitor binds to the active site of staphopain C at a ratio 1:1, and that the cysteine side chain is its target. This suggests the possibility of a wider use of the obtained biotinylated inhibitor to identify and titrate cysteine proteases in biological samples. As it was shown, the inhibitor binds covalently to the enzymes and thus, tagged proteins might be separated by SDS-PAGE and identified by blotting. This inhibitor can be an ideal and easy tool to obtain a probe to study cysteine proteases. Further research will involve kinetic studies of the obtained inhibitor with cysteine proteases.

## Figures and Tables

**Table 1:** Coupling reagents used for reaction with iodoacetic acid.

Sample No.	Coupling Reagent	Reaction Time (h)	Reaction Yield
1	COMU/DIPEA	2	31%
2	DIC	2	35%
3	DIC/HOBt	2	35%

## Supplementary Materials

The following are available online, Figure S1: NMR spectrum.
