# Supplementary material for: Synthesis of the Novel Covalent Cysteine Proteases Inhibitor with Iodoacetic Functional Group
Source: Molecules. 2020 Feb 13;25(4):813. doi: 10.3390/molecules25040813 (PMC7070521; doi:10.3390/molecules25040813)
Supplement: Supplementary file 1 [file molecules-25-00813-s001.pdf]

19-08-21-pmiel-iaa-leu-tyr-ahx-lys-bio-nh2.107.fid
Jagiellonskie Centrum Innowacji
Pracownia NMR
Probka: iaa-leu-tyr-ahx-lys-biotin-nh2

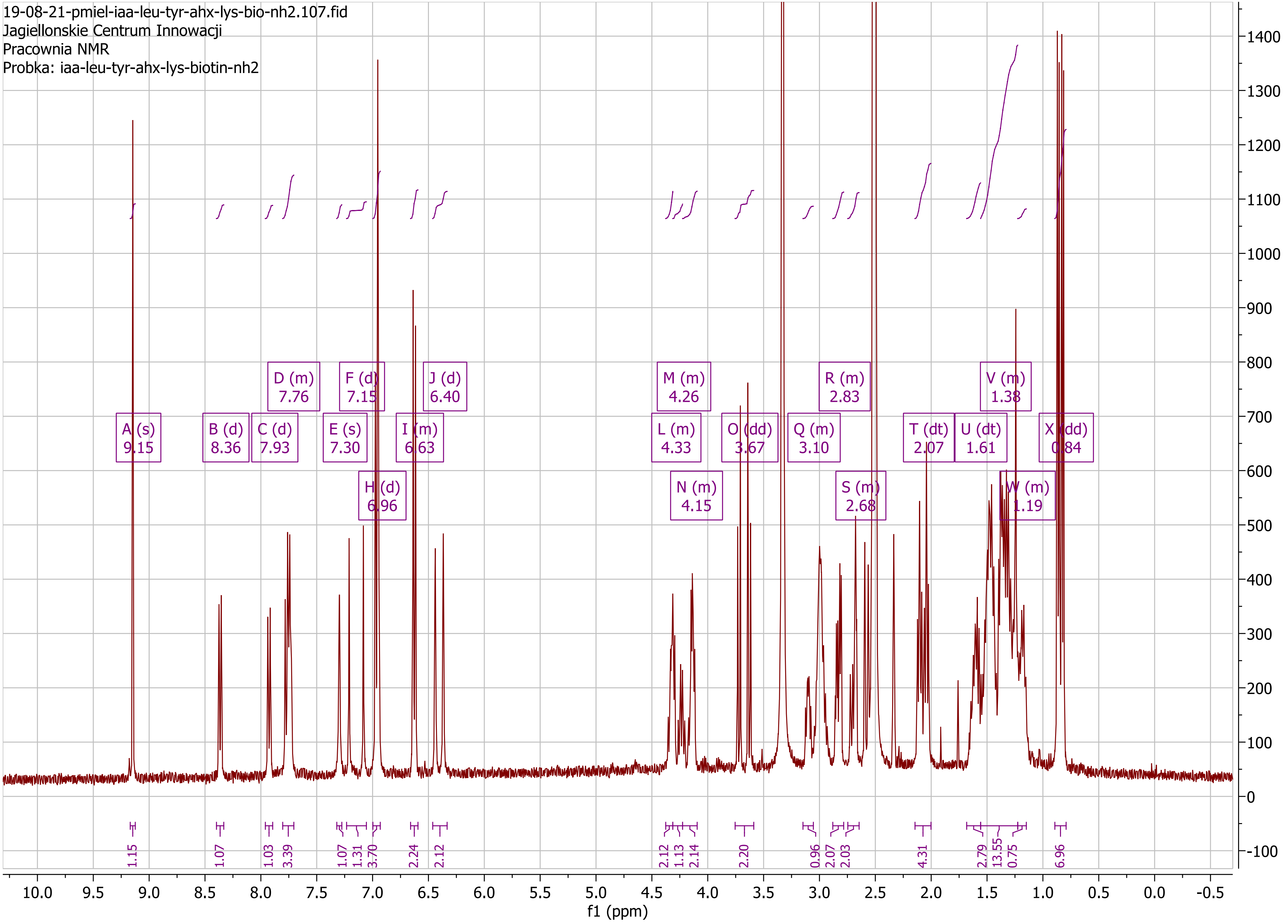

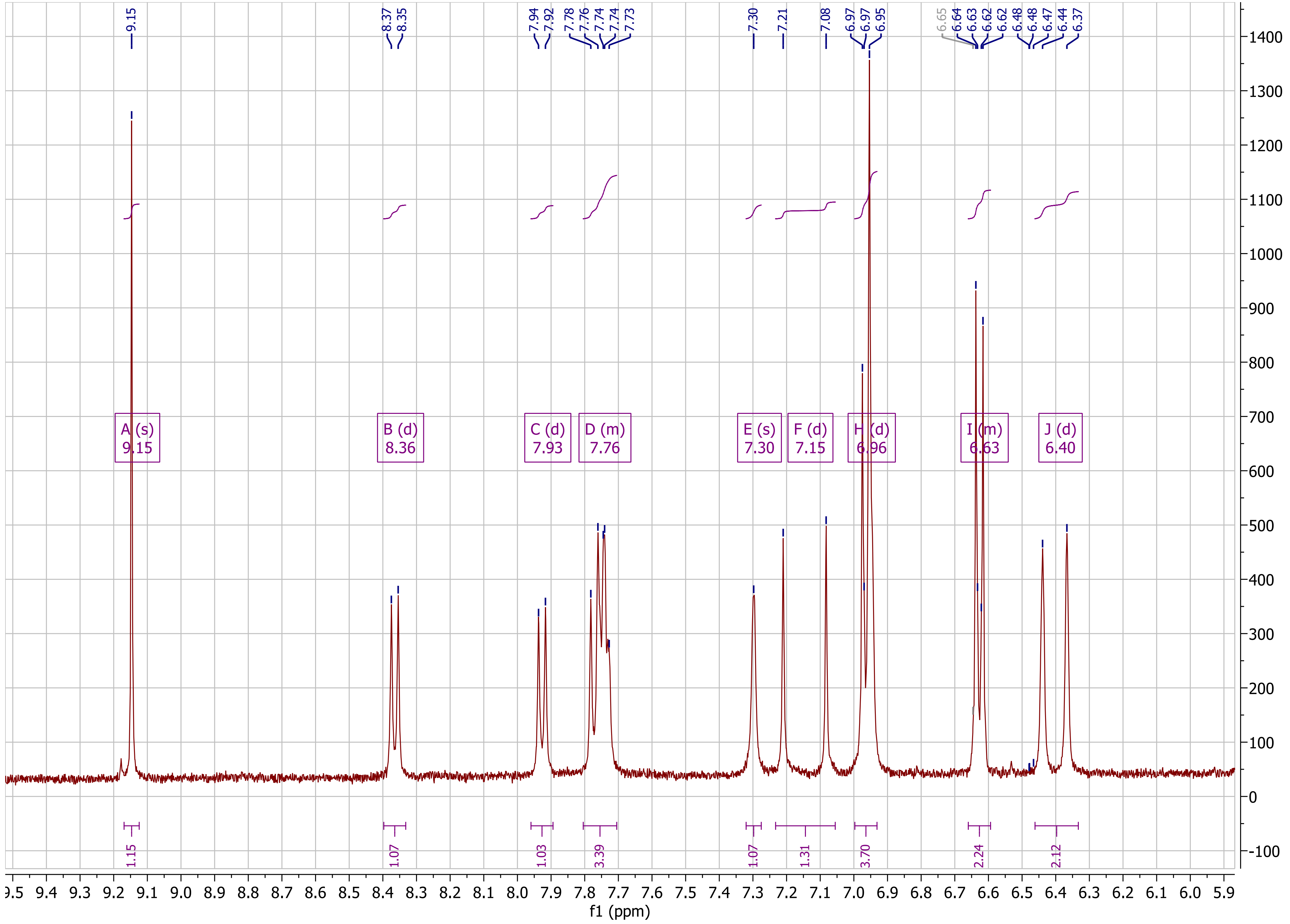
9.15
8.37
8.35
7.94
7.92
7.78
7.76
7.74
7.74
7.73
7.30
7.21
7.08
6.97
6.97
6.95
6.65
6.64
6.63
6.62
6.62
6.48
6.48
6.47
6.44
6.37
A (s) 9.15
B (d) 8.36
C (d) 7.93
D (m) 7.76
E (s) 7.30
F (d) 7.15
H (d) 6.96
I (m) 6.63
J (d) 6.40
1.15
1.07
1.03
3.39
1.07
1.31
3.70
2.24
2.12
f1 (ppm)

1H NMR (400 MHz, DMSO-d6) δ 9.15 (s, 1H), 8.36 (d, J = 8.1 Hz, 1H), 7.93 (d, J = 8.2 Hz, 1H), 7.80 – 7.70 (m, 3H), 7.30 (s, 1H), 7.15 (d, J = 51.1 Hz, 1H), 6.96 (d, J = 8.4 Hz, 4H), 6.66 – 6.59 (m, 2H), 6.40 (d, J = 28.9 Hz, 2H), 4.38 – 4.31 (m, 2H), 4.31 – 4.23 (m, 1H), 4.23 – 4.09 (m, 2H), 3.67 (dd, 2H), 3.15 – 3.05 (m, 1H), 2.88 – 2.78 (m, 2H), 2.75 – 2.65 (m, 2H), 2.07 (dt, J = 24.2, 7.5 Hz, 4H), 1.61 (dt, J = 13.2, 7.3 Hz, 3H), 1.56 – 1.23 (m, 14H), 1.23 – 1.15 (m, 1H), 0.84 (dd, J = 15.8, 6.6 Hz, 7H).
